# Attack of the clones: reproductive interference between sexuals and asexuals in the *Crepis* agamic complex

**DOI:** 10.1002/ece3.2353

**Published:** 2016-08-18

**Authors:** Evan Hersh, Jaime Grimm, Jeannette Whitton

**Affiliations:** ^1^ Department of Botany and Biodiversity Research CentreThe University of British Columbia6270 University BoulevardVancouverBritish ColumbiaCanadaV6T 1Z4; ^2^ Present address: Department of BiologyMcGill University1205 Dr. Penfield AvenueMontrealQuébecCanadaH3A 1B1

**Keywords:** Apomixis, eco‐evolutionary dynamics, interploidy crosses, interspecific pollen transfer, reproductive interference

## Abstract

Negative reproductive interactions are likely to be strongest between close relatives and may be important in limiting local coexistence. In plants, interspecific pollen flow is common between co‐occurring close relatives and may serve as the key mechanism of reproductive interference. Agamic complexes, systems in which some populations reproduce through asexual seeds (apomixis), while others reproduce sexually, provide an opportunity to examine effects of reproductive interference in limiting coexistence. Apomictic populations experience little or no reproductive interference, because apomictic ovules cannot receive pollen from nearby sexuals. Oppositely, apomicts produce some viable pollen and can exert reproductive interference on sexuals by siring hybrids. In the *Crepis* agamic complex, sexuals co‐occur less often with other members of the complex, but apomicts appear to freely co‐occur with one another. We identified a mixed population and conducted a crossing experiment between sexual diploid *C. atribarba* and apomictic polyploid *C. barbigera* using pollen from sexual diploids and apomictic polyploids. Seed set was high for all treatments, and as predicted, diploid–diploid crosses produced all diploid offspring. Diploid–polyploid crosses, however, produced mainly polyploidy offspring, suggesting that non‐diploid hybrids can be formed when the two taxa meet. Furthermore, a small proportion of seeds produced in open‐pollinated flowers was also polyploid, indicating that polyploid hybrids are produced under natural conditions. Our results provide evidence for asymmetric reproductive interference, with pollen from polyploid apomicts contributing to reduce the recruitment of sexual diploids in subsequent generations. Existing models suggest that these mixed sexual–asexual populations are likely to be transient, eventually leading to eradication of sexual individuals from the population.

## Introduction

Understanding the processes that govern the coexistence of close relatives has been a long‐time goal of evolutionary biology. Darwin (1859) first proposed that closely related species should have similar traits due to a shared evolutionary history, and this ecological overlap would intensify competitive interactions and limit co‐occurrence. This idea, termed the “competition‐relatedness hypothesis” (Cahill et al. 2008), or “phylogenetic limiting similarity hypothesis” (Violle et al. 2011) pervades the literature on species' co‐occurrence, but other processes may prevent the coexistence of close relatives. For example, Kuno's (1992) model proposes that negative reproductive interactions between species can lead to exclusion much more readily than competition over shared resources. Reproductive interference, the negative fitness effects resulting from interspecific mating attempts, has been reported in various plant (e.g., Gilissen and Linksens 1975; Armbruster and Herzig 1984; Galen and Gregory 1989; Harder et al. 1993; Runquist and Stanton 2012; Eaton et al. 2012; Takakura 2013; Nishida et al. 2013) and animal (e.g., Hettyey and Pearman 2003; Dame and Petren 2006; Valero et al. 2008; reviewed in Gröning and Hochkirch 2008) systems. Despite its recognition, reproductive interference has largely been overlooked as a general explanation for exclusive species distribution patterns (Kyogoku 2015), and its effects on community structure have rarely been considered (Gröning and Hochkirch 2008). Recently, however, there has been a renewed interest in the range of impacts of reproductive interference (Kyogoku 2015), from conservation biologists predicting the outcomes of biological invasions (Liu et al. 2007; Nishida et al. 2013) to behavioral ecologists mystified with interclass mating attempts (e.g., Antarctic fur seal and king penguin; de Bruyn et al. 2008).

Reproductive interference may be particularly impactful on plant species distributions, given that plant mating systems provide many avenues through which reproductive interference may act. Heterospecific pollen transfer has been shown to negatively impact seed set in a variety of ways: reduction in stigma receptivity (Waser and Fugate 1986), “stigma clogging” (Galen and Gregory 1989), inhibition of conspecific pollen tube growth (Thomson et al. 1982), “style clogging” (Brown and Mitchell 2001), and ovule wastage due to pollen incompatibility (Harder et al. 1993). Hybridization in plants is also widespread (Whitney et al. 2010), and while the fitness of hybrids varies (Rieseberg and Carney 1998), unfit hybrids (particularly early generation hybrids) have been reported in several studies (e.g., Heiser 1947; Grant 1966; Li et al. 1997).

Polyploidy produces derivatives that are likely to arise in sympatry with their diploid progenitors, and in such cases, interspecific pollen transfer is likely to be common and potentially costly. For example, triploid offspring from crosses between newly formed tetraploids and their diploid progenitors are often plagued by problems in endosperm formation and meiosis, resulting in inviable or unfit progeny (i.e., triploid block; reviewed in Köhler et al. 2010). Thus, when pollen transfer in sexual polyploid systems yields unfit offspring, there will be negative impacts on both polyploids and diploids, with the net effect likely to be most detrimental to the minority cytotype (Levin 1975). Although models suggest that there are restrictive conditions in which stable coexistence is possible, the most likely outcome of coexistence of ecologically equivalent diploids and tetraploids predict extirpation of polyploids or diploids (Fowler and Levin 1984; Felber 1991; Rodríguez 1996). These dynamics are believed to favor ecological divergence of polyploids from their diploid progenitors and play a key role in polyploid establishment and persistence (Grant 1971; Levin 1983; Ramsey and Schemske 1998; Rieseberg and Willis 2007).

In some plant groups, transitions to polyploidy are accompanied by transitions to apomixis (asexual seed formation), resulting in complicated “agamic complexes” (Babcock and Stebbins 1938) that include closely related diploid sexual species and an array of polyploid apomictic derivatives. Apomicts produce most or all of their seeds asexually, but, typically, still produce some viable pollen (Verduijn et al. 2004). Like sexual polyploids, apomictic polyploids may exchange pollen with their diploid progenitors, but the ovules of newly arisen apomicts will be at least partially reproductively isolated from their sympatric sexual progenitors. Apomicts typically bypass sexual reproduction by producing egg cells without meiosis, and embryos without fertilization (Savidan 2007). Because apomictic eggs are not being fertilized by pollen of mismatched ploidy, apomicts may avoid minority cytotype exclusion that typically limits sympatric establishment and persistence of new sexual polyploids (Cosendai and Hörandl 2010). Meanwhile, the pollen of apomicts can negatively affect the recruitment of co‐occurring sexual diploids, and it also has the potential to pass the apomictic trait on to the offspring from such crosses (De Wet 1968; Noyes and Rieseberg 2000; Berthaud 2001). As a result, when apomictic polyploids and sexual diploids co‐occur, sexuals are likely to be at a disadvantage. Sexual ovules appropriated by pollen from apomicts can produce polyploid and/or apomictic offspring, which reduce diploid sexual recruitment. Furthermore, if any of these mating attempts yield fertile apomictic offspring, these new hybrid apomicts could establish a new apomictic type, with the overall outcome that some progeny from sexual diploid mothers may end up contributing to the relative increase of apomicts at the expense of diploid sexual recruitment.

Although reproductive interference is not synonymous with hybridization (Kyogoku 2015), it can be costly if the hybrids are unfit or effectively lost from one or both of the parent's populations. When reproductive interference is asymmetrical or unidirectional (i.e., when one species exerts reproductive interference on another species), it reduces the recruitment of the species experiencing reproductive interference into the next generation; this shifts their relative abundances to favor the interfering species in a positive feedback loop, further increasing the amount of reproductive interference being exerted and eventually leading to exclusion (Kuno 1992; Nishida et al. 2013). In an agamic complex, polyploid apomictic pollen fertilizing diploid sexual ovules can yield apomictic progeny from sexual eggs, leading to the replacement/displacement of sexual morphotypes and redistribution of the complex. This unique combination of closely related microspecies that differ in ploidy and reproductive mode make agamic complexes valuable study systems for investigating the effects of reproductive interference. The effect of pollen from apomicts has been explored experimentally and theoretically (Maynard Smith 1978; Mogie 1992; Asker and Jerling 1992; Garani 2014), but its effect on naturally co‐occurring sexuals and asexuals has not been well investigated.

The North American *Crepis* agamic complex comprises seven distinct sexual diploids and a multitude of derivative apomictic polyploids, all of which occur at middle elevations, primarily in sagebrush communities of the Sierra Mountains and Great Plains regions of North America. Apomixis in *Crepis* is through apospory, in which the embryo develops from a somatic cell of the nucellus (Stebbins and Jenkins 1939). Studies of ovule development found that in all but one of the *Crepis* apomicts examined, the majority of ovules (>78%) develop aposporous embryos and that embryo development is often initiated before the flowers open, therefore precluding fertilization. Apomixis in *Crepis* is therefore autonomous – it does not require pollen for embryo or endosperm initiation (Stebbins and Jenkins 1939).

Phylogenetic analysis of plastid DNA variation (Sears and Whitton 2016) provides support for monophyly of most sexual diploids, with apomicts showing evidence of multiple polyploid origins. Apomicts and sexuals overlap in distribution in northern California and adjacent Oregon and Nevada, and in the Columbia Basin of central Washington and Oregon. Across the range of the complex, it is common for multiple morphologically distinct taxa to co‐occur locally. A survey of more than 100 sites found that roughly 40% have 2–8 co‐occurring “cyto‐taxa” (unique species and ploidy combinations). Phylogenetic data suggest that these are only occasionally the possible result of in situ origins of new polyploids. Rather, their unique plastid haplotypes indicate multiple colonization events at suitable sites (Sears and Whitton 2016). The only study of genetic variation within populations (a limited study of two sites each with three co‐occurring taxa) found evidence that at one site, co‐occurring apomicts comprised a very small number of multilocus genotypes (1–2 per taxon) consistent with very high levels of asexual reproduction (Whitton et al. 2008). At their second site, Whitton et al. (2008) found evidence of both clonality and recombination, and evidence of gene flow between two of the co‐occurring types. Using a randomization approach, Whitton et al. (in prep.) found that the co‐occurrence of apomicts across a set of >100 surveyed sites fit a model of random co‐occurrence, but that sexuals rarely co‐occur with other sexuals or with apomicts. Reproductive interference could account for this pattern, because of the strong asymmetry in the potential for reproductive interference to negatively impact sexuals. Given that most *Crepis* apomicts produce some viable pollen (Whitton et al., in prep.) and that the species have protracted and overlapping flowering periods and share pollinators, pollen flow from apomicts is likely to reduce recruitment of sexual diploid offspring either by reducing seed set (if crosses are incompatible) or by leading to the production of new hybrid polyploids.

In this study, our aim was to assess the potential for asymmetric reproductive interference from apomicts to contribute to local exclusion of sexuals by performing a crossing experiment in one of the few available sites where a sexual diploid co‐occurs with an apomictic polyploid. We predict that pollen from apomicts will reduce the relative potential for recruitment of sexual diploids by reducing seed set, seed quality, and/or by producing polyploid hybrids.

## Materials and Methods

### Study site

The locality that we studied is situated in Chelan County, Washington, on a dry slope above highway 97 on the south shore of Lake Chelan, just west of Chelan (Collection of C. Sears 4011; Lat. 47.8358, Long. ‐120.067; Sears and Whitton 2016). Fourteen samples from the population were previously characterized using flow cytometry and found to contain diploid individuals of *Crepis atribarba* subsp. *originalis* (henceforth *C. atribarba*; seven samples with 2C DNA content of 12.3‐13.9 pg) and high ploidy individuals of *C. barbigera* (6 individuals inferred to be 7x or 8x, with 2C DNA content of 46.1–52.0 pg). A single individual of *C. atribarba* had a DNA content consistent with pentaploidy (2C DNA = 32.7; ploidy estimates are based on mean diploid values for each taxon from a broader survey of the agamic complex; Sears 2011; Sears and Whitton 2016). Although the samples in the previous analysis were not randomly chosen, they nonetheless suggested that both taxa are common at this site and that most *C. atribarba* are diploid. Limited study of pollen viability in this population was consistent with overall trends in the complex: a single diploid individual had an estimate of 95% viable pollen, while the single pentaploid and octaploid individuals had 33% and 49% estimated pollen viability, respectively.

We surveyed the standing population on 20 May 2014 in order to determine the suitability of the site for assessing the effects of asymmetric reproductive interference. We identified individuals as either *C. barbigera* or *C. atribarba* and collected leaf samples from a total of 24 individuals in silica gel for subsequent flow cytometric analysis of ploidy, in order to confirm ploidy levels reported from earlier surveys (Sears 2011). At the time of this initial visit, most individuals of *C. atribarba* had open flowers and buds, while individuals of *C. barbigera* were not yet flowering. We noted but did not measure spatial structure in the distribution of the two taxa at the site. *Crepis atribarba* was more abundant at the western end of the site, and *C. barbigera* at the eastern edge, but nonetheless, the two species often occurred nearby one another, and fourteen of 31 *C. atribarba* plants used as mothers in our crossing experiment had at least one *C. barbigera* plant within a 3 m radius.

### Crossing experiment

Between 5th to 10th of June 2014, we conducted a crossing experiment aimed at determining the potential for pollen from the apomictic *C. barbigera* to sire seeds of the sexual diploid *C. atribarba*. On day 1, we assigned 31 *C. atribarba* to be maternal plants, selecting plants that had at least five unopened buds with which to perform crosses. Unopened flower heads were covered in fine mesh bridal veil and secured with twist ties to exclude pollinators. On day 2, we randomly assigned a minimum of two heads each as pollen recipients in crosses with diploid conspecific (diploid x diploid; DxD) and polyploid heterospecific (diploid x polyploid; DxP) pollen donors. At least one additional unopened head was covered to serve as an isolation control (I) used to confirm that the diploids are not apomictic (all diploids in the North American complex are reported to be sexual outcrossers, presumably with sporophytic self‐incompatibility; Stebbins and Jenkins 1939; Hughes and Babcock 1950; as found in other Asteraceae Hiscock 2000). Isolation treatments were also assigned to 10 *Crepis barbigera* individuals to confirm autonomous apomixis. On each maternal plant used in crosses, two additional heads that had finished flowering (indicated by closed phyllaries around withered corollas) were also covered to prevent seed dispersal and used as an open pollination treatment (Open; O), providing a measure of seed set under natural conditions.

We conducted crosses on 4 days (days 3–6), with individual heads receiving pollen on at least 2 days. On day three (the first day that covered buds opened), we began pollinations on the individual labeled X01 and ended with individual X31. On subsequent days, we started ten plants further down the sequence (e.g., at X11 on day 4), and then continued in numerical sequence from that plant through the full set of 31 maternal plants. We collected heads from pollen donors at the start of each day of crossing and replenished these after having performed crosses on 15 plants. We collected two heads from each pollen donor and placed these together into one of two bags per pollen source (i.e., two bags for diploid pollen donors and two for polyploid pollen donors). For each cross type performed on each maternal plant, we randomly retrieved two pollen donor heads (e.g., one head from each of the two “polyploid” bags) to reduce the probability that cross failure would result from sporophytic self‐incompatibility. We performed crosses by gently brushing each donor head against the exposed recipient heads for roughly 30 sec. In order to minimize the chance of contamination with the alternate pollen sources on the hands of the researcher, on each day of crossing, one researcher performed all DxD crosses, while the second researcher performed all DxP crosses. These roles alternated on each day of crosses to minimize the impact of researcher technique on seed set. Heads typically flowered over 2–3 days, beginning to wither after that time. To ensure that each head received at least 2 days of pollination, we removed from the experiment any heads that had not opened by day 5. During the course of the experiment, especially on the first two nights, some bags were apparently lost to deer browsing, but the deer learned to avoid the nylon mesh bags after this time. As a result of losses, some heads assigned as isolations were reassigned to crossing treatments. We deemed this preferable to losing power in the crossing experiment. As a result, we lack isolation controls for four maternal plants.

Following the completion of crosses, seeds were left to ripen and the bags were collected on 26 June 2014. Seed heads were dissected in the laboratory and categorized as either filled (dark colored and plump, presumed viable) or unfilled (unpigmented and flat, presumed inviable or unfertilized). After counting and sorting, all viable seeds from each mother were weighed together (to reduce measurement error associated with such small seeds) and the mass divided by the total number of filled seeds for each mother.

### Flow cytometry

Flow cytometry was performed to confirm the ploidy of 13 *Crepis atribarba* and 11 *Crepis barbigera* individuals. We estimated DNA ploidy (Suda et al. 2006; subsequently referred to as ploidy) from nuclear DNA content using a FACSCalibur II flow cytometer (BD Bioscience) located at the UBC Biomedical Research Facility. 10 mg of dried leaf tissue was “chopped” by reciprocating saw (Alexander et al. 2006) in 0.8 mL Otto I buffer (Otto 1992); Doležel & Gohde 1995) and 1 *μ*L mL^−1^ *β*‐mercaptoethanol before being filtered through a 30‐*μ*m screen. These nuclei suspensions were stored at 4°C for a minimum of 30 min. We prepared our three internal standards *Pisum sativum* (2C = 9.90 pg; Dolezel et al. 1992), *Secale cereale* (2C = 16.19 pg; Dolezel et al. 1998), and *Viccia fava* (2C = 26.90 pg; Dolezel et al. 1992) using the same methods. As our samples may have been of various ploidies (where a single standard would not have been sufficient), we prepared our standards separately instead of co‐chopping. Running the sample first individually allowed us to determine its approximate DNA content; this permitted the choice of an appropriate internal standard, which was then co‐run with the sample. Staining solution was prepared by adding 50 *μ*L propidium iodide and 100 *μ*L RNase (1 mg mL^−1^) per 800 *μ*L Otto II staining buffer. 100 *μ*L of sample, 50 *μ*L of standard, and 75 *μ*L of staining solution were mixed and let stain for at least 2 min before running through the cytometer.

Nuclei populations were monitored using FL2‐A histograms in BD CellQuest (BD, Franklin Lakes, NJ), and we analyzed the resulting CellQuest files using FlowJo vX.0.6 (Tree Star Inc., Ashland, OR). Nuclei peaks were isolated from background debris by gating scatter plots of FL2‐A by FL2‐W, and the mean position (channel number, CN) and coefficient of variation (CV) were calculated for each FL2‐A peak. Genome size (2C‐values) was calculated based on the linear relationship between sample and standard fluorescence intensities (Dolezel and Bartoš 2005). Only peaks with low coefficients of variation (CV < 8) were included in the dataset. Peaks with low nuclei counts (<500) were run separately (with standards run externally) and only included if the CV was low and the peak was easily identified as an appropriate DNA content (i.e., had a value that fell within the range of observed values in *Crepis*). The distribution of 2C values for low, intermediate, and high ploidy samples did not overlap, allowing ploidy classes to be assigned confidently.

Similar methods were used to assess the ploidy of seeds from open‐pollinated (O) and experimental crosses (DxD and DxP). We used the same methods as above, except the volumes were halved (as we used approximately half of the above‐mentioned biomass), and the grind time was increased from 30 sec to 90 sec. In order to keep similar ratio of biomass to buffer and maintain adequate volumes of nuclei solution, two seeds were used in each sample. We were able to determine the ploidy of both seeds in the sample by assessing the number of peaks. For example, if there were three separated peaks, we assumed that the two seeds had different ploidies (i.e., one peak from the standard, and one peak from each seed). We attempted a total of 60 seeds from DxD crosses, 61 seeds from DxP crosses, and 119 seeds from O crosses.

It should be noted that flow cytometric seed screening (FCSS) has been used in other taxa to assess reproductive mode by comparing the ratios of embryo: endosperm ploidy (e.g., Kao 2007; Hörandl et al. 2008). However, we were unable to utilize this method in our study because Asteraceae have ephemeral endosperm that is largely absent when seeds are fully mature (Noyes 2007).

### Statistical analyses

We used generalized linear models (GLM) and performed an analysis of deviance with quasibinomial errors (and a logit link function) to compare seed set between experimental (DxD and DxP) and control (O) crosses. We used a post hoc Tukey's HSD test to identify treatments with significant differences in seed set. We used linear models (LM) and performed an analysis of variance to compare seed mass between crossing treatments. We also tested for an effect of number of days of manual pollination on seed set in experimental crosses. Levene's test was used to check for homogeneity of variances, and the data were checked for normality. Seed mass was log‐transformed as the data proved to be non‐normally distributed.

All statistical analyses were performed in the R environment (R Core Team 2015), and the “car” package was used for ANOVA (Fox and Weisberg 2010).

## Results

### Seed set and seed mass

**Figure 1:**
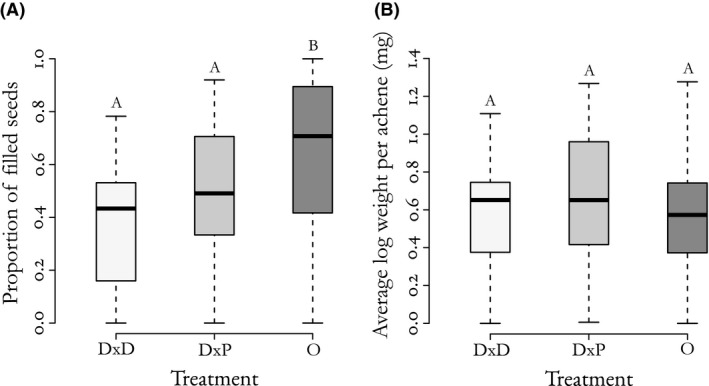
Median, quartiles, and outliers by treatment for reproductive traits after field crosses between *Crepis atribarba* and *C. barbigera*. Tukey's test results are shown as letters above box plots. (A) Proportion of filled seeds. Diploid x diploid treatment (DxD; *n* = 31, $\overset{¯}{X}$ =0.38 ± 0.10), diploid x polyploid treatment (DxP; *n* = 30, $\overset{¯}{X}$ =0.48 ± 0.11), open treatment (O; *n* = 30, $\overset{¯}{X}$ =0.64 ± 0.12). (B) Average log weight per filled achene (mg). DxD *n* = 24, $\overset{¯}{X}$ = 0.58 ± 0.14 mg; DxP *n* = 26, $\overset{¯}{X}$ = 0.66 ± 0.14 mg; O *n* = 27, $\overset{¯}{X}$ = 0.60 ± 0.13 mg.

Seed set differed among crossing treatments (*F*_2,87_ = 6.54, *P *=* *0.0023; Fig. 1A). Tukey's HSD test showed that while there was no difference between DxD and DxP crosses (*P *=* *0.555), open‐pollinated (O) crosses had higher seed set than both DxD (*P *=* *0.002) and DxP (*P *=* *0.037), indicating that the experimental crosses may have been pollen limited. Seed mass did not differ between crossing treatments (*F*_2,74_ = 0.462, *P *=* *0.63; Fig. 1B). There was a marginally nonsignificant interaction between number of days of pollination and treatment (*F*_1,57_ = 3.45, *P *=* *0.069).

### Ploidy determination

**Figure 2:**
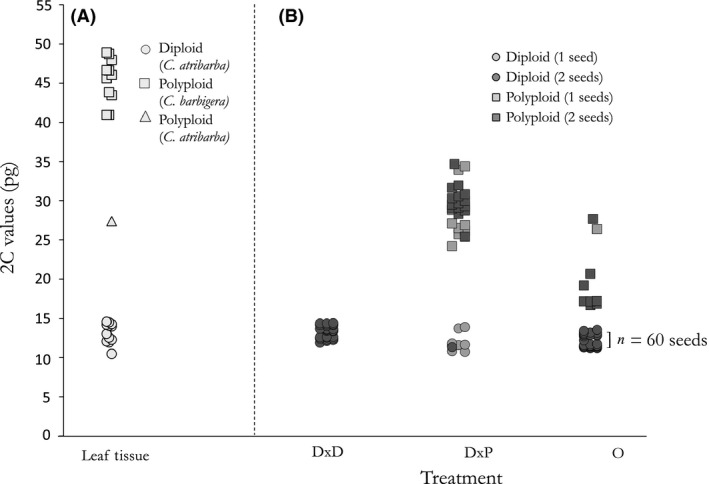
(A) Genome size (2C values) of tissue from adults identified as *C. atribarba* and *C. barbigera* in the field. *n* = 24. (B) Genome size (2C values) of seeds from field crosses between *Crepis atribarba* and *C. barbigera* by treatment. Diploid x diploid (DxD) treatment *n* = 50, diploid x polyploid (DxP) treatment *n* = 51, and open (O) treatment *n* = 77.

Comparison of flow cytometry estimates of DNA content of standing individuals against known ranges for diploids and polyploids of these taxa (Sears and Whitton 2016) confirmed that all but one sampled *C. atribarba* were diploid (2C range: 10.42 –14.53 pg; $\overset{¯}{X}$ = 13.12 ± 0.38 pg) and that *C. barbigera* individuals were of high ploidy (~7x–8x; 2C range: 40.83–48.77 pg; $\overset{¯}{X}$ = 45.32 ± 0.85 pg); a single sample identified as *C. atribarba* (Fig. 2A, 2C = 27.33 pg) was polyploid. The DNA content of this individual is intermediate between the mean diploid and high ploidy values, with the simplest interpretation being that it represents a hybrid (e.g., a pentaploid, from the union of tetraploid [reduced] pollen and haploid egg). Seeds from the experimental crosses were of various ploidies (Fig. 2B). Fifty seeds from DxD crosses, 51 seeds from DxP crosses, and 76 seeds from O crosses yielded useable data and were included in the dataset. All seeds from the DxD treatment were diploid, while the majority of seeds (40 of 51, 71.4%) from the DxP treatment were of intermediate ploidy (2C range: 24.17–34.59 pg; $\overset{¯}{X}$ = 29.19 ± 0.54 pg), confirming that hybrid seeds can be formed from cross‐pollination. The remaining 11 seeds of DxP crosses fell into the expected range for diploids (2C range: 10.7–13.86 pg; $\overset{¯}{X}$ = 11.85 ± 0.34 pg). These seeds are most likely the product of facilitated selfing (Mentor effect, see below). Open‐pollinated seeds (O) were mostly diploid (62 of 76 seeds, 81.6%), but a total of 17 seeds from four maternal plants were intermediate, indicating that hybrid seeds can be formed under natural conditions. Any seeds from the O treatment with 2C values that varied more than 10% from the *C. atribarba* tissue mean (see above) were classified as of intermediate ploidy.

## Discussion

The aim of our study was to determine whether interspecific pollen flow from apomicts can exert reproductive interference on the sexual species in the *Crepis* agamic complex. It is important to note that in considering the role of reproductive interference in this system, our focus is to understand whether the presence of an apomict can reduce recruitment of the sexual diploids, and therefore potentially lead to their eventual local extirpation. We interpret the production of polyploid hybrids as evidence of asymmetric reproductive interference on diploids because hybrids reduce the relative production of diploid offspring.

We found that hybrid seeds are readily formed between diploid sexual *C. atribarba* and apomictic polyploid *C. barbigera* individuals; to our knowledge, this is the first crossing study conducted in the North American *Crepis* complex. Most of the seeds resulting from crosses between the polyploid (~7x‐8x) apomicts and 2x sexuals were polyploid and approximately intermediate (~5x); this confirms their hybrid origin (DxP treatment; Fig. 2B), which we interpret as most likely resulting from reduced pollen from the apomicts (~4x) fusing with haploid eggs of the sexual. We found no difference in seed set or seed mass between the crossing treatments, suggesting that apomictic pollen is not reducing the early stages of reproductive fitness of sexual diploids in this population. Moreover, the production of apparently healthy seeds suggests that pollination by apomicts can usurp ovules of *C. atribarba* mothers (DxD vs. DxP treatments; Fig. 1) and potentially reduce the number of diploid *C. atribarba* individuals available for recruitment into the next generation. Given that *C. barbigera* can exert such asymmetrical reproductive interference on *C. atribarba*, it is possible that their existence in sympatry is unsustainable in the long term.

While the frequency of hybrid seed production in our experimental crosses is likely to be artificially high because we did not use mixed outcrossed pollen loads, we have some evidence that hybrids are produced naturally as well. We found a moderate proportion (18.4%) of polyploid (~5x) seeds in open‐pollinated heads, demonstrating that interspecific pollen transfer can also result in hybrid formation under natural conditions (O treatment; Fig. 2B). In addition, natural pentaploid individuals were found in a previous census (1:14 individuals; Sears and Whitton 2016) as well as our own (1:24 individuals). We note however that the standing individuals surveyed in both our sample and that of Sears and Whitton (2016) were nonrandom and modest in number, and in our case, we intentionally did not sample individuals with intermediate morphology, so these results should not be taken as an estimate of the frequency of hybrids in the standing population. Nevertheless, the fact that we found hybrids in relatively small censuses indicates that viable hybrids can be formed in this population.

While we were unable to test the viability of the seeds (due to difficulties with germination in the lab), we found comparable seed mass between DxP (mostly hybrid) and DxD (diploid) seeds. Seed size has been shown to be a determinant of germination success in *Crepis tectorum* (Andersson 1996), and this relationship may apply in our species as well. Our data suggest that hybrid and nonhybrid seeds are equally provisioned and could have similar success establishing on the landscape. It should be noted that the formation of hybrid seeds in our experiment indicates the presence of reproductive interference, whether the seeds have higher, equal, or lower viability than diploid seeds. However, the strength of reproductive interference will differ: if hybrid seeds are inviable, reproductive interference would act only by reducing the proportion of diploid seed formed. If at least some hybrids are viable, as we suspect based on the fact that some were found in censuses of standing individuals, these could compete for suitable sites and contribute to reproductive interference in later generations. In this case, reproductive interference could impact both original apomicts and sexuals.

Our flow cytometry data suggest that the hybrids are most likely pentaploids. Their reproductive success will depend on whether they are sexual or apomictic. Plants with odd ploidies typically have reduced fertility due to imbalanced meiotic products and the formation of aneuploid gametes (Comai 2005). If the hybrids in our study include sexual pentaploids, we expect that their fertility will be low. On the other hand, as previously mentioned, apomixis can be transmitted via pollen (De Wet 1968; Noyes and Rieseberg 2000; Berthaud 2001), so it is possible that some proportion of the hybrid offspring may be partially or fully apomictic. Fully apomictic offspring will bypass aforementioned meiotic imbalances in ovules and produce asexual seed unhindered. Therefore, this scenario is also expected to lead to reproductive interference by reducing the relative representation of sexual diploids in the standing population.

Although not previously documented in *Crepis*, we were not surprised to detect a modest number of diploid seeds (11 of 51) from diploid–polyploid crosses. Interploidy crosses in both sexual and apomictic polyploid systems are known to sometimes yield selfed seeds due to breakdown of self‐incompatibility (SI) systems (i.e., mentor effects; Richards 1986). These mentor effects have been shown to occur in several otherwise self‐incompatible sexual members of agamic complexes (Tas and van Dijk 1999; Mráz 2003; Brock 2004). While we have no direct evidence that our diploid seeds are the product of selfing (as opposed to diploids of hybrid origin), the fact that the pollen donors are 7x‐8x supports this conclusion, because such polyploids would be expected to produce haploid pollen only rarely, if at all. Although the production of selfed seeds reduces the production of hybrids, we expect selfed seeds of outcrossers such as *C. atribarba* to be subject to inbreeding depression (Glemin et al. 2001; Busch 2004), and therefore these selfed seeds may constitute another negative outcome of reproductive interactions. It is unclear whether mentor effects normally play a big role in this system. In a mixed sexual–apomictic population, sexual stigmas will most likely receive a mixture of pollen from apomicts, self pollen, and sexual‐outcross pollen. Mixed self and outcross pollen loads typically lead to significantly higher outcross paternity and considerable abortion of self‐sired seeds (Montalvo 1992). This may cause low self‐paternity following natural pollination in mixed populations; a study explicitly aimed at disentangling these effects would be a useful follow‐up.

Our results differ in some ways from findings in other systems in which interploidy crosses have been conducted. Crosses between different ploidy levels often cause endosperm incompatibilities, leading to aborted seeds (Levin 2002). For example, Hörandl and Temsch (2009) conducted crosses between sexuals and apomicts in the *Ranunculus auricomus* complex. The majority of their crosses resulted in aborted seeds, and the remainder was either formed by mentor effects or cross‐fertilization; these processes are thought to have greatly reduced the potential for introgression of apomixis. However, we did not find a large proportion of aborted seeds in our crosses, suggesting that endosperm incompatibilities are not affecting seed viability, and thus not reducing the potential strength of reproductive interference.

Taken together, our results are consistent with the potential and action of asymmetrical reproductive interference, with negative consequences for the sexual diploid *C*. *atribarba* due to interspecific pollen transfer from co‐occurring *C*. *barbigera*. Although we were only able to investigate the early stages of hybrid formation, the high production of filled hybrid seeds from experimental crosses together with documentation of naturally occurring seeds and mature individuals of intermediate ploidy are congruent with our predictions for reproductive interference. Although limited to a single population, our results provide evidence for a previously undocumented mechanism that can serve as a barrier to long‐term coexistence between sexual and apomictic types in the *Crepis* agamic complex, and suggest that asymmetric reproductive interference with negative impacts on sexual populations may account for the overall observation of low co‐occurrence of sexuals with apomicts, despite frequent and apparently random co‐occurrence of apomicts (Sears and Whitton 2016). While further surveys will be important for understanding frequency and consequences of hybrid formation at sites where sexuals and apomicts co‐occur (both in *Crepis* and in other sexual–apomictic complexes), existing models suggest that even modest levels of asymmetric or unidirectional gene flow from apomicts to sexuals can lead to local extirpation of sexuals (Mogie et al. 2007; Mogie 2011).

The particulars of the *Crepis* agamic complex allowed us to investigate an unusual form of asymmetrical reproductive interference. While the conditions allowing reproductive interference to occur in our system are quite specific to agamic complexes in which apomicts maintain pollen function, they add to a growing body of work that suggests that reproductive interactions may play an important role in limiting coexistence of close relatives. This phenomenon has potential conservation implications given that several recent studies have found evidence that non‐native plant species can displace native congeners via reproductive interference (Brown and Mitchell 2001; Brown et al. 2002; Matsumoto et al. 2009; Takakura and Fujii 2009; Nishida et al. 2011, 2013). Multiple examples of asymmetrical reproductive interference have also been found, all predicting the eventual displacement of the species most affected by the interaction (Burgess et al. 2007; Takakura et al. 2008; Takakura 2013; Runquist and Stanton 2012). These examples provide evidence that costly reproductive interactions have the potential to influence plant community structure by promoting ecological sorting, species turnover, and floral divergence among species (Eaton et al. 2012).

## Conflict of Interest

None declared.

## Supporting information


**Data S1.** Model coefficients and relevant values (F, P, df, etc.) for statistical models used. Output from R.
